# Supplementary material for: Evidence supported by Mendelian randomization: impact on inflammatory factors in knee osteoarthritis
Source: Front Med (Lausanne). 2024 May 28;11:1382836. doi: 10.3389/fmed.2024.1382836 (PMC11165061; doi:10.3389/fmed.2024.1382836)
Supplement: Supplementary file 1 [file Data_Sheet_1.ZIP › data/GCST90274833_5e6/833.leaveoneout.pdf]

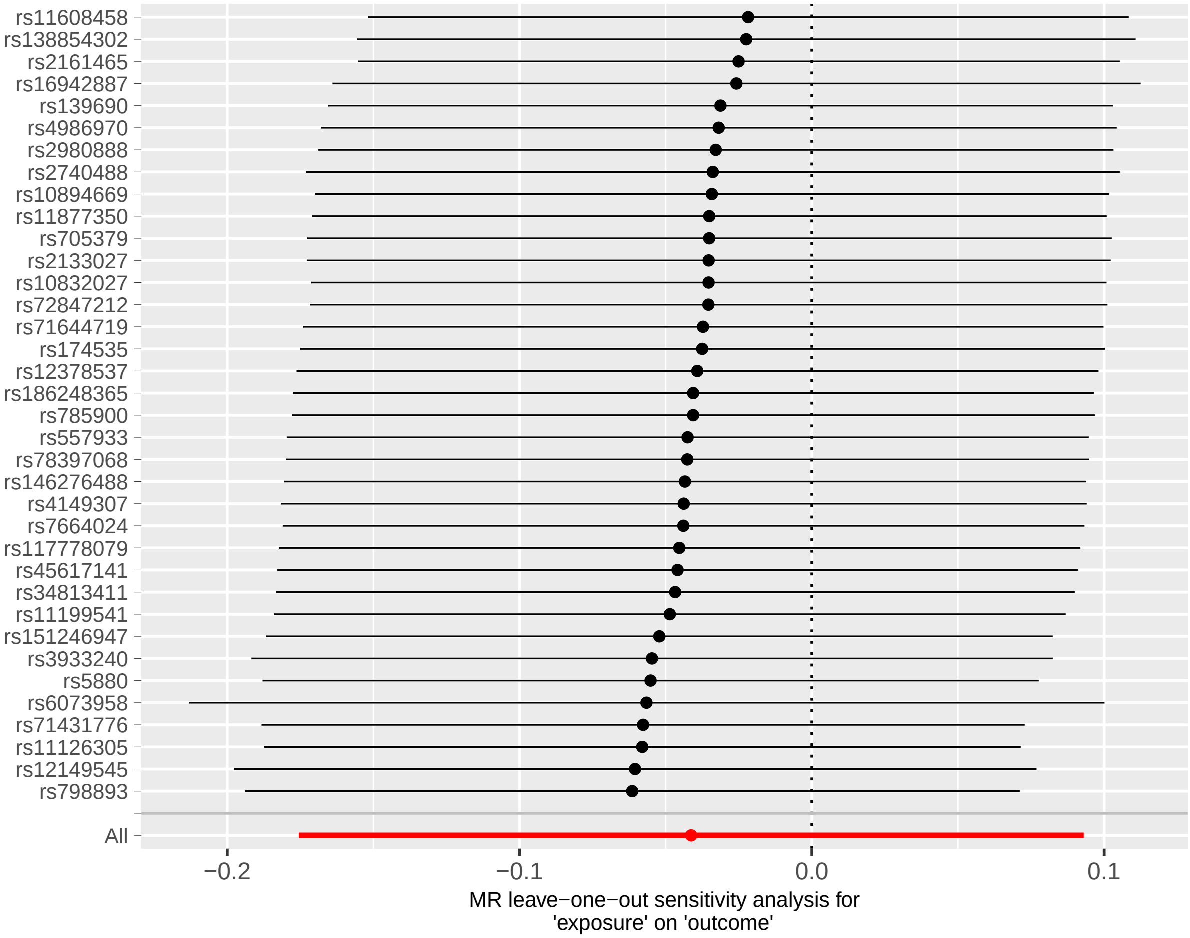
rs11608458
rs138854302
rs2161465
rs16942887
rs139690
rs4986970
rs2980888
rs2740488
rs10894669
rs11877350
rs705379
rs2133027
rs10832027
rs72847212
rs71644719
rs174535
rs12378537
rs186248365
rs785900
rs557933
rs78397068
rs146276488
rs4149307
rs7664024
rs117778079
rs45617141
rs34813411
rs11199541
rs151246947
rs3933240
rs5880
rs6073958
rs71431776
rs11126305
rs12149545
rs798893
All
−0.2
−0.1
0.0
0.1
MR leave−one−out sensitivity analysis for
'exposure' on 'outcome'
